# Cationic Porphyrins as Antimicrobial and Antiviral Agents in Photodynamic Therapy

**DOI:** 10.3390/cimb45120612

**Published:** 2023-12-06

**Authors:** Inga O. Savelyeva, Kseniya A. Zhdanova, Margarita A. Gradova, Oleg V. Gradov, Natal’ya A. Bragina

**Affiliations:** 1 Institute of Fine Chemical Technology, MIREA—Russian Technological University, Vernadsky Prospect 86, Moscow 119571, Russia; inga.saveleva.96@mail.ru (I.O.S.); zhdanova_k@mirea.ru (K.A.Z.); n.bragina@mirea.ru (N.A.B.); 2 N.N. Semenov Federal Research Center for Chemical Physics, Russian Academy of Sciences, Kosygin Street 4, Moscow 119991, Russia; m.a.gradova@gmail.com

**Keywords:** viruses, bacteria, photodynamic inactivation, photosensitizers, singlet oxygen, porphyrins

## Abstract

Antimicrobial photodynamic therapy (APDT) has received a great deal of attention due to its unique ability to kill all currently known classes of microorganisms. To date, infectious diseases caused by bacteria and viruses are one of the main sources of high mortality, mass epidemics and global pandemics among humans. Every year, the emergence of three to four previously unknown species of viruses dangerous to humans is recorded, totaling more than 2/3 of all newly discovered human pathogens. The emergence of bacteria with multidrug resistance leads to the rapid obsolescence of antibiotics and the need to create new types of antibiotics. From this point of view, photodynamic inactivation of viruses and bacteria is of particular interest. This review summarizes the most relevant mechanisms of antiviral and antibacterial action of APDT, molecular targets and correlation between the structure of cationic porphyrins and their photodynamic activity.

## 1. Introduction

Despite the rapid development of medicine, pathogenic microorganisms are still a threat to human health [1]. Such microorganisms include bacteria, including antibiotic-resistant bacteria, as well as viruses. The emergence of multidrug-resistant (MDR) bacteria has become a challenge for modern healthcare systems. The seriousness of the problem is evidenced by the discovery of new strains of pathogens that exhibit resistance to all the known types of antimicrobial drugs and is caused by the irrational use of antibiotics by people [2]. Thus, according to a report by the World Health Organization, none of the 43 antimicrobial drugs that were in clinical trials in 2020 managed to fully solve the problem of bacterial resistance [3]. Considering the problem of overuse of antibiotics, it should be mentioned that only 7% of COVID-19 cases were accompanied by bacterial infections, while more than 70% of patients received antibiotics even in the absence of any clinical indication [4]. In addition, the increasing interaction of humans with nature inevitably leads to the emergence of a number of pandemics caused by microorganisms. Thus, the SARS-CoV-2 coronavirus pandemic turned out to be one of the most serious pandemics in recent decades. Every year scientists record the emergence of three to four previously unknown types of viruses that are dangerous to humans, the total number of which is more than 2/3 of all the newly discovered human pathogens [5,6]. In addition, there is also an increase in viral resistance to the antiviral drugs due to their high mutation rate. It is obvious that to combat newly emerging and resistant forms of already known infections, it is necessary to develop safe and highly selective compounds with a wide spectrum of action [7].

Antibiotics and antivirals are the most extensively used chemotherapeutical treatments for infectious diseases. The fast rate of mutation by bacteria or viruses helps them to survive and develop resistance to current drugs. Development of new antibacterial or antiviral drugs is the most common strategy to overcome drug resistance. The widespread use of antibiotic drugs has led to the emergence of so-called “superbacteria”, which are resistant to virtually all types of antibiotics. The major mechanisms of drug resistance include reducing drug internalization, overexpressing drug efflux pumps, sequestering entered drugs, modifying drug targets, as well as the formation of biofilms [8,9,10,11]. At the same time, administration of antibiotics leads to a number of undesirable side effects: a negative impact on the normal microflora of the organism, nephro-, oto-, neurotoxic and other effects. In addition, some classes of antibiotics, for example, glycopeptide, have a pseudoallergic effect—the so-called “red face syndrome” due to the release of histamine from mast cells.

Currently, alternatives to traditional antibiotic therapy approaches are being developed. Such approaches include the use of immunobiological drugs (vaccines, antibodies, probiotics and immunostimulants), biological defense agents (bacteriophages, phagolysins and artificial bacteriophages), as well as new chemotherapeutic agents based on peptides (antimicrobial peptides), antibacterial nucleic acids, antibiofilm compounds and some others [12,13,14,15]. Despite a wide variety of new alternative approaches, there have been no striking breakthroughs in the treatment of infections for more than a decade [16]. Many of the new methods are at the experimental research stage or have even been developed only theoretically [17]. Phage therapy is considered as one of the most promising methods in terms of clinical potential and ease of use [18]. Nevertheless, the appearance on the market of ready-to-use drugs to replace antibiotics is still questionable. The significance of immunostimulants, which are already used for disease prevention and as additional means of therapy, is unclear for clinical practice, so they do not look like a full-fledged substitute for antibiotics.

A promising approach to solving the problems associated with the fight against microorganisms is antimicrobial photodynamic therapy (APDT). Traditionally, photodynamic therapy (PDT) has been used in the treatment of malignant and non-malignant neoplasms [19]. Since the mid-1990s, PDT has been used to kill microbes because bacterial cells multiply quickly, similar to malignant tumor cells [20]. Antibacterial photodynamic therapy is a non-antibiotic process that causes bacterial cell death in the presence of photosensitizing drugs, light energy of the appropriate wavelength and molecular oxygen [21]. APDT is a promising and inexpensive potential alternative to conventional chemotherapy treatment [10,11]. Interest in APDT as a method for treating infectious diseases has significantly increased in the last decade. The applicability of this method has been established in relation to Gram-positive and Gram-negative bacteria, fungi, protozoa, as well as a number of enveloped and non-enveloped viruses [20,21,22,23]. For a range of infections, APDT is a promising and inexpensive alternative to the traditional chemotherapeutic treatment. This approach is already used in medical practice to treat a number of infections and for surface disinfection [24,25,26]. The use of PDT is unlikely to cause microorganism resistance, since apoptosis under the influence of reactive oxygen species (ROS) occurs through a nonspecific mechanism of inhibition, in contrast to antibiotics, the action of which is aimed at certain enzymes and key cellular processes.

In the field of photodynamic inactivation of viral particles, there are currently several main biomedical and clinical areas: the treatment of local infections caused by the herpes simplex virus and human papillomavirus with the approved drugs and photosensitizers (PSs) [27,28], technologies for blood product disinfection [29,30,31] and the development of self-sterilizing materials and surfaces [32,33,34]. In addition, this technology has been proposed for food processing, since a number of natural photosensitizers, for example, curcumin or riboflavin, can be safely added to food products [35,36,37].

Numerous studies show that tetrapyrrole compounds and their analogues, which include natural and synthetic porphyrins, chlorins and bacteriochlorins, due to their tunable physical and chemical characteristics, are promising not only for the treatment of cancer, but also for use in APDT [24]. Tetrapyrrole macrocyclic compounds, as a class of biologically active substances, combine in their molecules a set of unique photophysical properties (intense absorption in the visible and near-IR regions, intense fluorescence, efficient singlet oxygen generation, etc.) with high chemical and thermal stability, and wide possibilities for chemical modification of the macrocycle [38]. To date, it has been established that positively charged agents based on porphyrins and their analogues are among the most effective PSs for APDT. PSs with cationic groups are not able to effectively penetrate the mammalian cell membranes, for this reason they are unsuitable agents for antitumor therapy. However, the presence of positive charges in their structure is crucial when targeting the membrane of bacteria, especially Gram-negative ones. It is positively charged PS molecules that are most often used to increase the light sensitivity of Gram-negative bacteria. In addition, a promising biological target for cationic porphyrins is the negatively charged DNA molecule.

Establishing the dependence between the structure of photosensitizing agents and their antimicrobial action is the most important task for improving the APDT method. This review examines the available data on the current state of research on photodynamic inactivation of bacteria, mammalian viruses and bacteriophages using cationic porphyrins. Particular attention is paid to the possible mechanisms of photoinactivation, molecular targets and factors influencing the process of viral inactivation, as well as the current developments in terms of treating Gram-positive and Gram-negative bacteria, their limitations, and future perspectives.

## 2. Principles of Antimicrobial PDT

Traditionally, PDT has been used for cancer treatment [39,40,41,42,43]. The introduction of a PS, its selective accumulation in tumor tissues and subsequent exposure to light of a certain wavelength leads to photodynamic reactions that produce ROS and free radicals. These reactive particles cause oxidative damage in tumors, leading to their death and subsequent replacement by normal tissues. In the 1990s, there was an increasing number of reports on the use of PDT to inactivate Gram-positive bacteria [20]. It was found that bacteria are more sensitive to ROS compared to mammalian cells, which makes the use of APDT for the treatment of infectious diseases possible [23].

### 2.1. Mechanisms of Photodynamic Action

**Figure 1:**
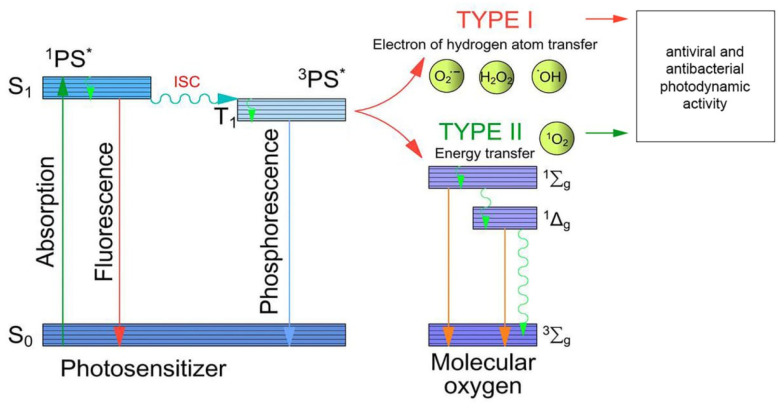
The mechanism of photodynamic action.; *PS in the excited state.

After absorbing light quanta, the PS molecules turn to the excited singlet state (^1^PS). A subsequent transfer to the lower energy but longer-lived triplet state (^3^PS), from which ROS are generated, can occur through intersystem crossing. The lifetime of the triplet state allows the PS to interact with the neighboring molecules. Type I photodynamic reactions are characterized by deactivation of the PS excited state due to electron transfer to the surrounding biomolecules (Figure 1). This results in the formation of free radicals, as well as anionic radicals of the PS and substrate. The common final substrate for type I reactions is molecular oxygen, leading to the formation of superoxide anion radicals (O_2_^•−^), hydroxyl radicals (^•^OH) and hydrogen peroxide. The initiated cascade of homolytic reactions causes oxidative stress, leading to the biochemical destruction of cells [44]. As oxygen runs out, the type I reaction mechanism becomes predominant when the amount of free molecular oxygen is limited. However, the most common type of photodynamic reaction is type II, where photoexcitation energy is directly transferred from the triplet PS to molecular oxygen resulting in singlet oxygen generation. Both mechanisms can operate simultaneously, and their relative contribution depends on the structure of the PS, its concentration and the oxygen concentration [45,46]. Tetrapyrrolic PSs are mostly characterized by type II photoreactions. At the same time, several studies have reported evidence of the greater sensitivity of Gram-negative bacteria to type I reactions [47].

### 2.2. The Mechanisms of Photodynamic Antibacterial Action

**Figure 2:**
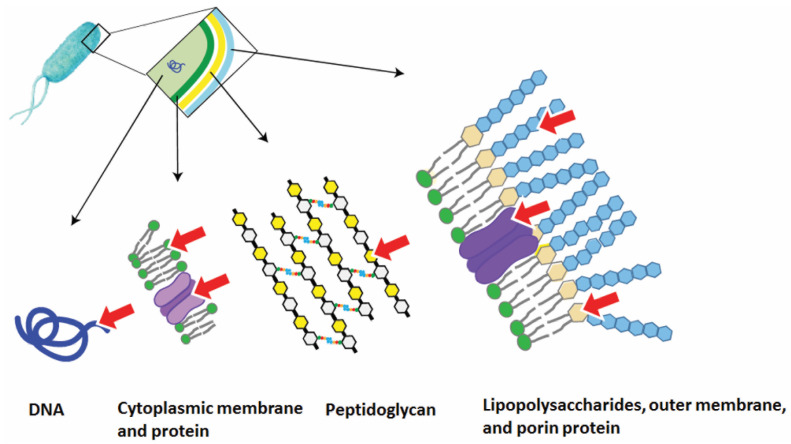
Overview of APDT targets in bacterial cells, indicated by a red arrow [39].

When carrying out APDT in vitro, a PS is added to the bacterial sample, which leads to its photosensitization. Under irradiation (400–800 nm) the resulting ROS cause oxidative stress in the bacteria (destruction of lipids, amino acid cross-links) [48,49]. ROS have a very short lifetime (10^−6^–10^−9^ s) and the diffusion distance of singlet oxygen in the cell is approximately 10–20 nm [50,51], so the main factor affecting the efficiency of photodynamic effects on microorganisms is the binding of PSs to their targets. It should be noted that photodynamic treatment methods are local: the photodynamic effect is observed only in the irradiated area containing PS in the presence of oxygen; therefore, their application is limited to the impact on the specific area of infection. It has been shown that there are two main types of molecular targets of PS for APDT: external (cytoplasmic membrane, cellular components) and internal targets (respiratory complexes, metabolic enzymes and nucleic acids) (Figure 2) [52,53,54]. Based on the experimental data, there are three main molecular mechanisms of the destructive photodynamic effect of the PS on bacterial cells:

1. The PS accumulates in the intercellular space, generates reactive oxygen species, which can subsequently diffuse into the target cells, causing their oxidative damage;
2. The PS binds to the structural elements of the bacterial cell due to hydrophobic or Coulombic interactions and transfers its energy to the intracellular biomolecules. This leads to the formation of ROS and the destruction of pathogenic tissues;
3. The PS independently penetrates the cell and binds to the intracellular targets, for example, polyanionic macromolecules, such as DNA [55].

### 2.3. The Mechanisms of Photodynamic Antiviral Action

The mechanism of photodynamic inactivation (PDI) of viruses is similar to that of bacteria:

1. The accumulation stage is similar to that of bacteria;
2. When affecting viral targets, ROS (generated via type I or type II mechanisms) can have a damaging effect on the viral nucleic acids, capsid proteins, as well as on the receptor proteins and lipoprotein envelope lipids for large viruses [56];
3. The negatively charged phospholipids of the virus envelope may play a decisive role in electrostatic interactions with the PS.

Several studies have also shown that PDI probably affects only certain stages of the virus life cycle [57]. Thus, a detailed study of the interaction of viral particles with photosensitizers at the different stages of viral production was carried out in the work of R. Neris [58]. Fluorescence images of viruses treated with 1,1′-dioctadecyl-3,3,3′,3′-tetramethylindodicarbocyanine (DiD) were presented in this work. DiD only emits fluorescence when the viral envelope fuses with the endosomal membrane during virus endocytosis. Treatment of the DiD-stained viruses with the PS and light stimuli led to the fluorescence quenching, indicating that viruses cannot fuse to the endosomal membrane. Transmission electron microscopy can also provide information about the condition of viral particles. Thus, after the PDT procedure, viral particles exhibit irregularities in particle structure resulting in symmetry loss, a tendency of viral particle aggregation as well as the appearance of a projection on the surface of the virus.

The photodynamic effect does not depend on the specific interaction between the PS and receptor; hence, this “nonspecificity” of photodynamic damage is one of the most important advantages of this method. Given the high genetic variability of viruses, this non-target mechanism of action will be least likely to cause drug resistance in the viral target. However, despite the current advances in APDT against viruses in vitro, mass treatment of patients or inactivation of viruses in biological media such as blood products has not yet been implemented. Viral nucleic acids can also be one of the targets for APDT. Some PSs can penetrate the virus envelope and intercalate into their DNA/RNA. Such interaction is especially effective in the case of cationic PSs. Oxidative transformations destroy DNA, leading to its fragmentation, single-strand breaks and cross-linking with proteins. ROS were shown to prevent viral replication and reduce their infectivity due to the DNA damage [59]. In particular, guanine molecules are susceptible to oxidative damage with the formation of 8-oxo-7,8-dihydroguanine as one of the main products of type I photodynamic reactions [60].

Generally, the structure of the used PS plays a crucial role in its inactivation capability. It has been established that anionic photosensitizers have a weak antiviral effect compared to cationic photosensitizers. In addition to phospholipids, the photodynamic effect of cationic PSs may also extend on the surface of viral envelope glycoproteins, since viral replication and virulence are controlled by several glycoproteins that play important roles in the process of virus entry into the host cell as well as cell-to-cell spreading [61]. Thus, a noticeable reduction in the time of virus inactivation can be achieved in the case of using cationic PSs as compared to anionic PSs, due to the stronger electrostatic interaction of positively charged PS molecules with phospholipids and the effect of this PDT agent on viral glycoproteins. The distribution coefficient in the octanol/water system is another important parameter that affects the PSs photodynamic activity since the viral envelope is characterized by a low polarity [62]. Consequently, more lipophilic compounds will have a higher affinity for the viral envelope, which should increase the likelihood of its binding to the virus [63].

Considering the available publications in this area, it can be concluded that there is no universal PS or chemical class of PSs that is best suited for PDI of viruses, and its efficiency always depends on the specific application area and the viral target. In recent publications, amphiphilic tetrapyrrole derivatives have been considered as the lipid-targeted PSs, and large enveloped viruses as a suitable target for such PSs [56,64]. In general, it should be noted that photodynamic effects on bacterial cells or viral particles do not cause the development of pathogen resistance to APDT treatment, since photodynamic inactivation of pathogenic microorganisms is completely nonspecific [65].

### 2.4. Photosensitizers for APDT

The most important elements for photodynamic therapy are photosensitizers. For the successful use of PDT, photodynamic agents should possess certain characteristics: a high degree of chemical purity; thermal stability; minimal dark cytotoxicity; water solubility or penetration into tissues. The absorption spectra of PSs and other substances in the body, such as melatonin, hemoglobin or oxyhemoglobin, should not overlap. Also, photodynamic agents should have an intense absorption peak in the red and near-infrared spectral regions (from 650 to 800 nm), since the absorption of single photons with a wavelength above than 800 nm does not provide sufficient energy for the transition of molecular oxygen into the singlet state.

**Figure 3:**
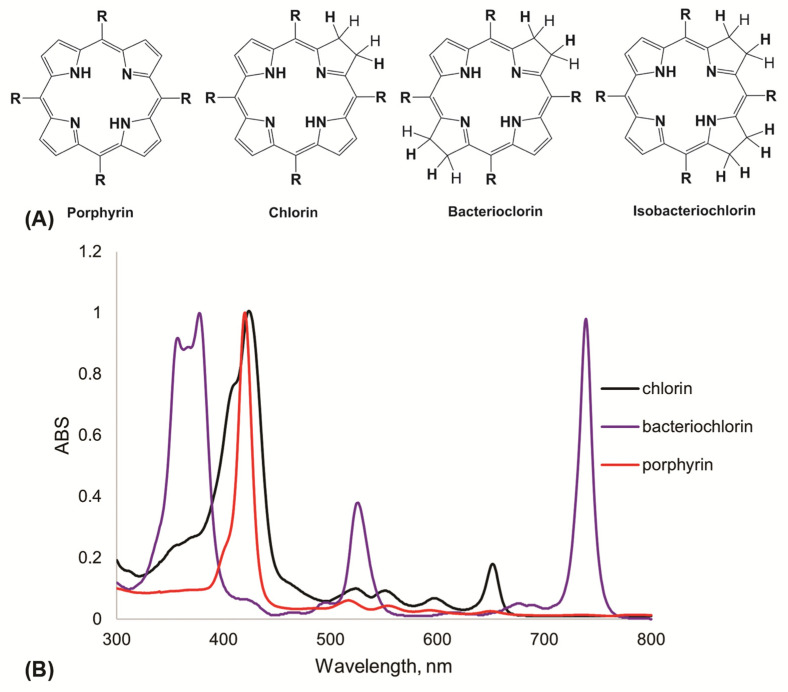
(**A**) Chemical structure of photosensitizers based on porphyrins and their derivatives; (**B**) normalized electronic spectra of porphyrin, chlorin and bacteriochlorin.

One of the most popular agents for APDT are synthetic porphyrins and their analogues [66,67]. Figure 3A shows the structures of synthetic meso-aryl-substituted porphyrins, chlorins, bacteriochlorins and isobacteriochlorins. All these compounds differ in their spectral characteristics. Porphyrins are first generation PSs. Porphyrins are distinguished by intense absorption within 410–420 nm (Soret band) and four non-intense absorption bands in the region of 500–700 nm. However, this class of compounds is characterized by chemical stability, high extinction coefficients (~500,000 M^−1^ cm^−1^) and singlet oxygen quantum yields (~0.5–0.7). Chlorins, bacteriochlorins and isobacteriochlorins belong to the second generation of PSs and are characterized by intense absorption in the region of 600–750 nm, but less stability, and when exposed to atmospheric oxygen they are susceptible to oxidation into porphyrins.

Among the existing classes of PSs, the safest for surrounding cells are the second generation PSs absorbing light in the IR region, widely used for antitumor PDT purposes [68]. However, these compounds also have a number of disadvantages—insufficient photostability, high cost, complexity of chemical modification. All these factors complicate the widespread introduction of IR PS in APDT of topical infections. On the other hand, cationic derivatives of porphyrins are more stable to oxidation, cheap to obtain and easily enough subjected to chemical modification—the developed chemistry of tetrapyrroles has, in its arsenal, effective approaches and methods of their preparation. Due to their chemical availability, porphyrins can serve as model compounds in elucidating the molecular mechanisms of antimicrobial action of the tetrapyrrole class.

### 2.5. Antibacterial and Antiviral Activity of Cationic Porphyrins

The well-developed chemistry of tetrapyrrole compounds makes them universal starting “platforms” for the design of new types of PSs for APDT. For several decades, porphyrin derivatives with positive charges have been used to increase the photosensitivity of Gram-negative bacteria by improving the interaction between PSs and the bacterial cell membrane through electrostatic interactions [69,70]. In the course of numerous studies, it was found that the charge of the PS turned out to be the main factor determining the effectiveness of photosensitization. Moreover, the degree of bactericidal effect is explained by the different structure of the bacterial cell wall: the presence of an outer membrane makes Gram-negative bacteria more resistant to photoinactivation than Gram-positive bacteria due to the presence of an additional outer membrane [66,71]. Cationic PSs can achieve effective interaction with negatively charged cell wall components, leading to higher photoinactivation efficiency compared to anionic or neutrally charged compounds. The number of charges and their distribution in the porphyrin macrocycle play an important role in the antimicrobial activity of PSs against Gram-negative bacteria. The effectiveness of cationic porphyrins against *E. coli* increases with the increasing number of positively charged groups, so porphyrins containing three or four positive charges in the molecule are the most effective PSs [72].

#### 2.5.1. Cationic Porphyrins Based on Tetrapyridylporphyrin (TMPyP) and Its Derivatives

**Figure 4:**
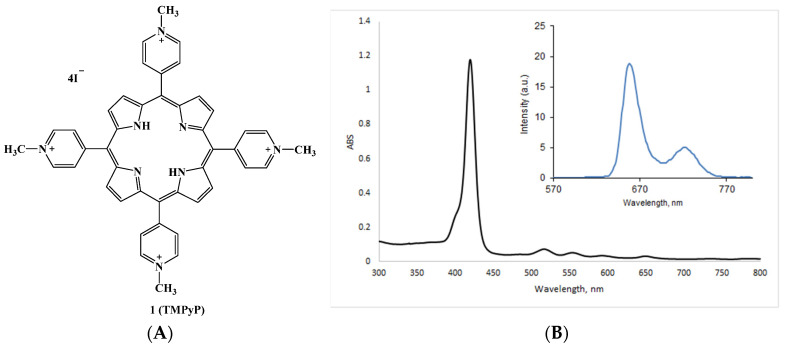
(**A**) Chemical structure of TMPyP tetraiodide; (**B**) UV-vis and fluorescence (insert) spectra of the TMPyP.

One of the most common cationic porphyrins is meso-tetra(4-N-methylpyridyl)porphine **1** (TMPyP) [73,74] in the form of tosylate or iodide salt (Figure 4A). The absorption spectrum of this compound contains one intense Soret band (420 nm) and four low-intensity Q-bands (518, 554, 585 and 630 nm), and TMPyP is characterized by a high quantum yield of singlet oxygen (Φ_Δ_ = 0.7). Its fluorescence spectrum in aqueous solution has two distinct peaks in the red region (between 649–655 and 715–720 nm, Figure 4B).

Previously, compound **1** was actively studied as a PS for PDT, particularly for the treatment of localized neoplasms [75], creation of porphyrin-loaded nanoparticles for APDT [76], ligands for G-quadruplex binding [77,78], and protein binding studies [79,80]. To date, TMPyP and its derivatives are actively studied as antimicrobial agents. TMPyP was the first porphyrin used in the studies of antibacterial activity, which revealed that meso-substituted cationic porphyrins can inactivate not only Gram-positive (*Enterococcus seriolicida*, *S. aureus*), but also Gram-negative bacteria such as *Vibrio anguillarum* and *E. coli* [81,82]. Valduga et al. demonstrated that *E. coli* are effectively inactivated by visible light after incubation with TMPyP, and the phototoxic activity of this porphyrin is mainly caused by disruption of the enzymatic and transport functions of the outer and cytoplasmic membranes [83].

**Figure 5:**
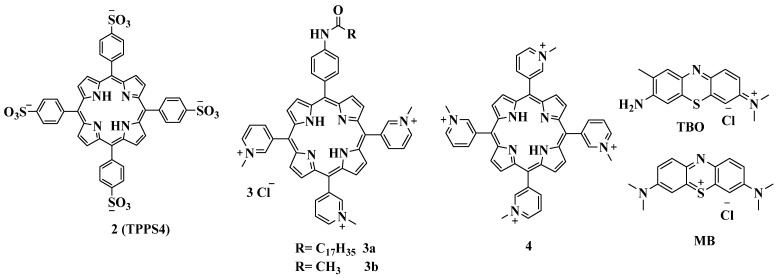
Chemical structure of compounds **2**–**4a**,**b**.

Donnelly et al. analyzed the potential treatment of lung infections caused by a clinical strain of Gram-negative *P. aeruginosa* with APDT using TMPyP and toluidine blue (TBO) [84]. It was shown that maximum toxicity (>99%) of TMPyP tetratosylate against clinical strains of *P. aeruginosa* was achieved at a concentration of 2.5 mg/mL; higher concentrations (5 mg/mL) were required to inhibit bacteria in biofilms. The work of Guterres et al. demonstrated that porphyrins with positive charges are more efficient PSs against *Mycobacterium massiliense* and *Mycobacterium fortuitum* than the negatively charged tetrasulfonatophenylporphyrin **2** (TPPS4). Compared to its anionic analogue **2** (Figure 5), cationic TMPyP **1** had a high yield of singlet oxygen, sufficient photostability, and a low level of aggregation in an aqueous solution. Irradiation with white light reduced the minimum inhibitory concentration (MIC) of TMPyP by more than 100-fold, while the MIC of TPPS4 decreased only by half. It was also noted that the effectiveness of using certain porphyrins as PSs for APDT is closely related to their peripheral charge and, therefore, solubility in physiological media [85].

The Lesar group concluded that *L. pneumophila* was sensitive to photodynamic inactivation with all the cationic porphyrins tested, with compound **3a** (Figure 5) being the most effective one, since it caused membrane damage and cell death at the lowest dose. Its photobactericidal activity exceeded the activity of methylene blue (MB) by approximately 1000 times. Amphiphilic porphyrin **3a** has three positive charges for binding to the bacterial cell through electrostatic interactions with the negatively charged lipopolysaccharides, and also has a long alkyl chain, a highly lipophilic moiety that facilitates its penetration through biological membranes. Among the porphyrins studied, tetracationic *meso*-tetra(3-N-methylpyridyl)porphin **4** turned out to be an even a more effective bacterial inhibitor than tricationic porphyrin **3b** [86].

One of the mechanisms of bacterial resistance is the formation of biofilms. A biofilm is a complex structure consisting of an exopolysaccharide matrix, proteins, lipids, DNA, RNA, ions and water. This complex structure complicates penetration of the antimicrobial drugs [87,88,89]. The study [90] aimed to evaluate the cytotoxicity, synergistic effect, antimicrobial and antibiofilm activity of water-soluble tetracationic porphyrins **1** and **4** against *P. aeruginosa*. When irradiated with white light for 120 min, the best values of minimal inhibitory (MIC) and minimal bactericidal concentrations (MBC) were obtained using TMPyP porphyrin in micromolar concentrations. It was also found that when TMPyP is used together with the β-lactam class antibiotic imipenem, a synergistic effect is observed. Porphyrin, when exposed to light, prevents operation of β-lactamase enzymes, enzymes that modify aminoglycosides, thereby reducing manifestation of bacterial resistance mechanisms to the commercial antimicrobial drugs. However, an antimicrobial drug can also improve the effect of photoinactivation by increasing the permeability of the outer membrane of bacteria to the PS molecules [11,91]. An effective reduction in the formation of biofilm by *Pseudomonas aeruginosa* was shown, and atomic force microscopy revealed that treatment of bacteria with TMPyP solutions followed by irradiation leads to the deformation of their cell wall.

The planar porphyrin macrocycle, containing four pyrrole nitrogen atoms, is well suited for binding metal ions to form metal porphyrins. It is known that the triplet state of diamagnetic metals, compared to the paramagnetic ones, has a longer lifetime and, accordingly, a higher quantum yield of singlet oxygen [92,93]. The introduction of a metal into the porphyrin cavity, along with the changes in peripheral substituents, can have a significant impact on the photophysical properties and photodynamic activity of the PSs studied. From this point of view, peripheral coordination complexes of Pt(II) or Pd(II) and porphyrins are of interest. Among the advantages of these compounds, one can highlight their ability to actively generate ROS [94,95]. In addition, such porphyrins are quite stable in solutions (DMSO and buffer solutions) and do not exhibit aggregation and photobleaching under irradiation [96].

**Figure 6:**
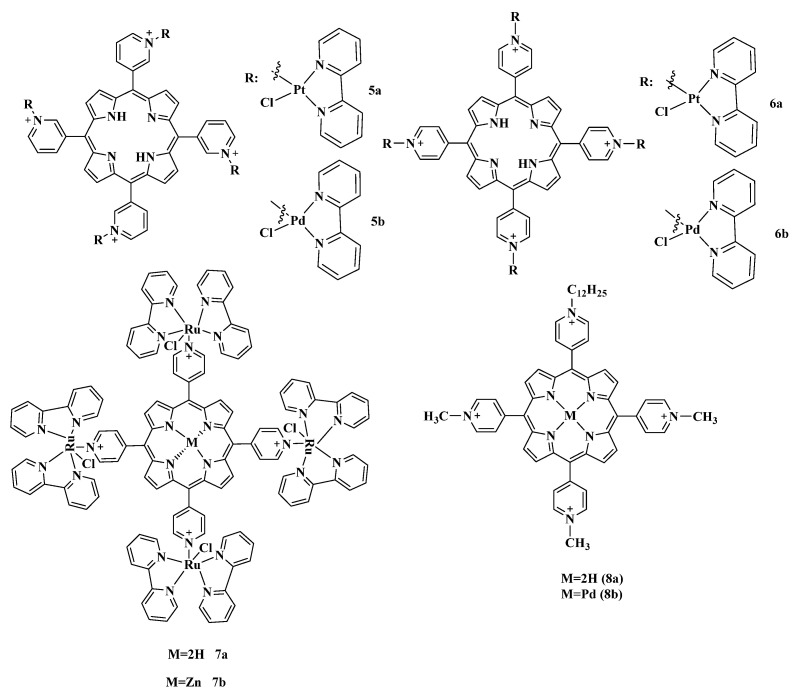
Chemical structures of the porphyrin metal complexes.

Thus, preparation of the porphyrin metal complexes can be an effective strategy for increasing PS efficiency. Iglesias et al. investigated antimicrobial photoinactivation using tetracationic porphyrins with peripheral bipyridyl complexes of platinum (II) and palladium (II) **5**–**6a**,**b** against the strains of fungal dermatophytes [97], rapidly growing strains of mycobacteria (RGM) [98] and a number of Gram-negative bacteria [96] (Figure 6). It was shown that in relation to the fungal strains *T.rubrum* and *M.canis*, meta-isomer 5a with platinum at the periphery of the macrocycle had the greatest activity when irradiated with white light. Meta-isomer with palladium at the periphery (compound **5b**) showed a significant antimicrobial effect against Gram-negative bacteria *E. coli*, *Klebsiella pneumoniae*, *Pseudomonas aeruginosa* and Gram-positive *S. aureus*, as well as mycobacteria. An important conclusion of this work was the fact that the activity of meta-substituted porphyrins turned out to be significantly higher compared to the para-substituted compounds **6a**,**b**. The authors associate the lower activity of para-substituted isomers with their reduced solubility in an aqueous environment, and thus a greater tendency to aggregation, which significantly reduces the efficiency of these PSs [99,100].

In the same group, the antibacterial and antibiofilm properties of conjugates of TMPyP and cisplatin, one of the most commonly used chemotherapeutic agents in the treatment of testicular, ovarian, etc., cancer were studied [101,102]. The clinical use of cisplatin is known to be limited by serious side effects such as nephrotoxicity, gastrointestinal toxicity, ototoxicity, and neurotoxicity [103]. Cisplatin has already been tested as an antibiofilm agent in *P. aeruginosa* biofilms because it can prevent DNA repair (required for the development of antimicrobial resistance) and penetrate the microbial biofilm better than other antimicrobial agents [104]. It was established that meta-isomer of the conjugate with cisplatin turned out to be the most effective PS, since its MIC and MBC in *P.mirabilis* upon irradiation decreased by four and two times, respectively, compared to dark conditions. In a biofilm inhibition test, meta-isomer at MIC and ½ MIC concentrations reduced the biofilm by 40 and 36%, respectively, under dark conditions. When using a white light source, the biofilm inactivation efficiency doubled: 83% at MIC and 61% at ½ MIC.

Ruthenium(II)-based compounds are of particular interest in biology and medicine due to their therapeutic potential as anticancer and antimicrobial agents. The work [105] reports on the photodynamic inactivation of *Salmonella enterica* using cationic porphyrin **7a** and its Zn(II) complex **7b**, containing peripheral Ru(II)-bipyridyl complexes (Figure 6). For porphyrin **7b**, the quantum yield of singlet oxygen was higher compared to free-base **7a**, which determined its better antibacterial effect. Zinc ions promote spin-orbital interaction, and hence, increase the probability of intersystem crossing.

**Figure 7:**
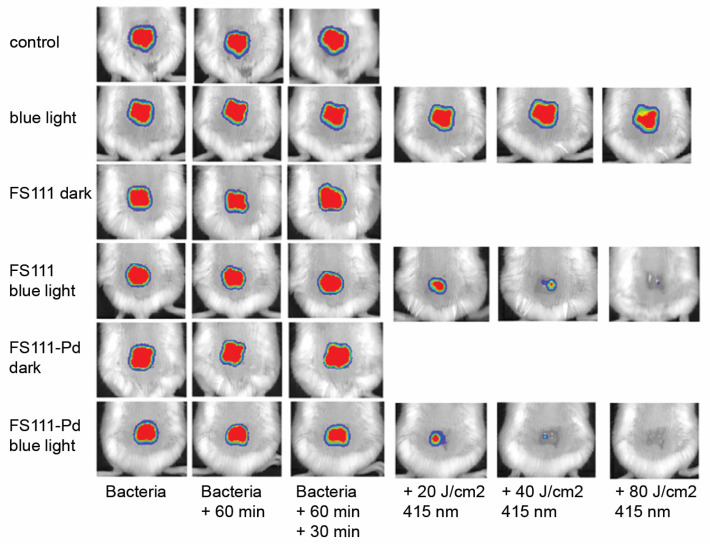
PDT in vivo of mice with *E. coli* wound infections. Panel of representative bioluminescence images captured from mice undergoing PDT with **8a** or **8b** and increasing fluences (20, 40 and 80 J/cm^2^) of 415 nm light. Copyright 2018 WILEY [106].

A high photoinactivating activity in vitro and in vivo was exhibited by new PSs based on unsymmetrically substituted TMPyP **8a**,**b** (Figure 7) [106]. An increase in the degree of amphiphilicity of this compound is achieved by the inclusion of a single dodecyl residue. Both compounds, free-base **8a** and its palladium complex **8b**, showed significant activity at nanomolar concentrations (10 nM), with the use of **8b** giving a 3log reduction in *S. aureus* strain. *E. coli* required increasing the dose of the drugs to 500 nM. In an in vivo study of a localized infection model with an *E. coli* strain, both compounds were shown to effectively reduce the bioluminescent signal in a dose-dependent manner under blue light excitation (405 nm), with **8b** preventing relapses for 6 days. Figure 7 show in vivo bioluminescence images of mice *E. coli* wound after PDT procedure.

It has previously been reported that photoinactivation of bacteria with PSs having a zinc (II) ion in the tetrapyrrole core results in severe damage to the lipid bilayer, oxidation of membrane proteins, and inactivation of respiratory complexes, which contributes to a decrease in ATP synthesis aerobically [107]. Consequently, bacteria are deprived of energy, the production of reducing equivalents (NADH, NADPH) is reduced, disrupting the natural cellular defense against oxidants, entailing oxidative damage in target molecules. In the following work, the authors remarked that the four main criteria for the PSs for APDT efficiency are: (1) the presence of a positive charge in the porphyrin macrocycle (four positive groups); (2) the presence of a central metal atom (Zn(II)); (3) the presence of hydrophobic peripheral functional groups; (4) high values of singlet oxygen quantum yields. A series of PSs with different peripheral substituents in the pyrrole ring were synthesized [108]. A number of TMPyP derivatives with hydrophilic oxyethyl group, hydrophobic butyl or allyl substituents and differing by the presence of zinc atoms in the macrocycle, were obtained. The quantum yields of singlet oxygen formation were quite high for the studied PSs (0.77 ± 0.04 for metal-free pyridylporphyrins and 0.89 ± 0.04 for metalloporphyrins). The structure of substituents and their position in the pyridyl ring did not significantly affect the efficiency of singlet oxygen formation. The zinc complex of tetrabutylpyridylporphyrin had the highest activity, for which effective photoinactivation of Gram-negative (*E. coli K-12*, *Salmonella typhimurium G-38*) and Gram-positive (*S. aureus*, *S. epidermidis*) bacteria, at a rather low concentration of 0.7–2 g/mL, was observed.

In [109], comparative studies of the photoactivity towards *S. aureus* bacteria of indium complexes for the neutrally charged compound **9a** and cationic **9b** (Figure 6) and their conjugates with magnetic Ag/CuFe_2_O_4_ nanoparticles were carried out. When studying photophysical and photochemical parameters, complexes **9a**,**b** themselves and their conjugates with nanoparticles showed a relatively high quantum yield of singlet oxygen in aqueous media. The authors note that when studying the antimicrobial activity of the resulting compounds, complex **9b** and nanoparticles based on it showed higher cytotoxicity, in contrast to the neutrally charged compound **9a**.

The antimicrobial activity of silver and various materials based on it is well known [110,111]. The work [112] reports an assessment of the efficiency of photoinactivation of a number of mycobacteria by Ag(II) complexes of the cationic TMPyP, **10a** and the anionic sulfoporphyrin **10b**. It was noted that the tetracationic porphyrin derivative in solution in a nanomolar concentration, coordinated with a silver (II) ion, has a high singlet oxygen quantum yield (Φ_∆_ = 0.81), which made it possible to effectively photoinactivate the strains of non-tuberculous mycobacteria. AFM studies of the bacterial damage revealed the formation of cavities in the membrane after treatment with a porphyrin solution under white light irradiation. In addition, changes in nanomechanical and electrostatic properties were observed: the adhesion force between the membrane of microorganisms and the extracellular environment, which plays an important role in the attachment of bacteria to surfaces and the formation of biofilm, decreased. When studying the oxidative mechanism of action of the tetracationic PS, the formation of hydroxyl radicals ^•^OH and superoxide anion O_2_^•−^ was recorded, which indicates the type I photochemical reactions.

The authors of [113] demonstrated photoinactivation of non-enveloped bacteriophage MS2 of the genus Levivirus within the family Leviviridae using TMPyP and proposed a possible mechanism for its inactivation. Bacteriophage MS2 is often used as a model organism because of its similarity in size and morphology to some human viruses [114]. The A-protein of MS2 is a major target of chemical oxidants and a potential target for photoinactivation. The amino acids histidine and tryptophan located in the middle of the A-protein are the most sensitive to the action of ^1^O_2_ and may be responsible for the rapid rate of loss of antigenicity. Photoinactivation of the virus by singlet oxygen causes aggregation of MS2 particles and cross-linking of virus envelope proteins. TMPyP at a concentration of 0.2 µM inactivated MS2 within 1 min after irradiation. The authors used antibodies specific to the sequences of four selected antigenic regions of the A-protein to establish the mechanism of action of the PS. Specific antibodies against antigenic sites 1 and 3 failed to detect A-protein after 10 min of PDI, and antibodies against antigenic sites 2 and 4 failed to detect A-protein even after 1 min of PDI, which corresponds to the rate of light-induced inactivation of MS2 in the presence of TMPyP. From the data obtained, it was hypothesized that singlet oxygen could cause loss of antigenicity of both the site that is attached to the viral RNA genome inside the capsid and the site located in the β-sheet domain of the A-protein, which may be one of the specific sites responsible for attaching MS2 to the bacterial pilus and delivering its genome inside the host. It is also likely that oxidation of the most singlet-oxygen-sensitive histidine and tryptophan, located almost in the middle of antigenic sites 2 and 4, respectively, contributes to the rapid rate of antigenicity loss.

**Figure 8:**
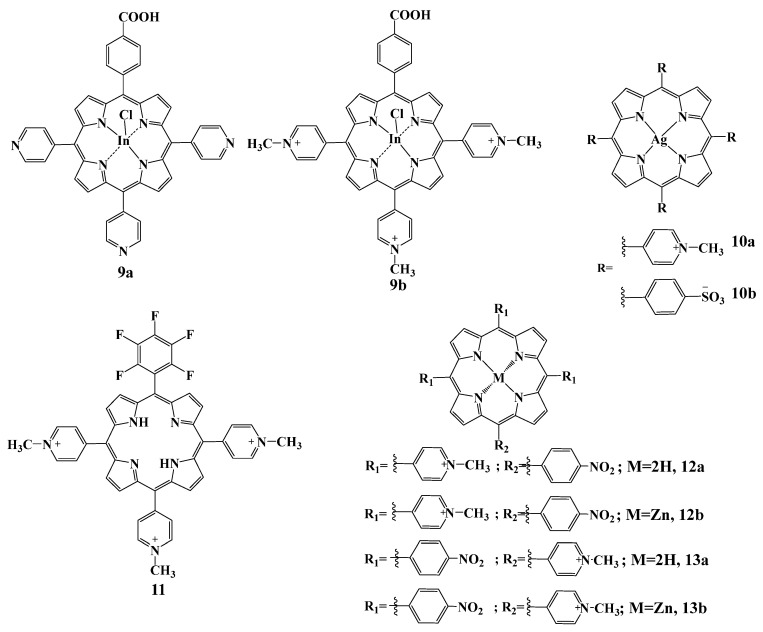
Chemical structures of the compounds **9**–**13**.

Liliana Costa and co-authors [115] studied the photodynamic activity of fluorine-containing tricationic porphyrin **11** (Figure 8) in relation to the model virus target—T4-like bacteriophage. Compound 10 was shown to possess significant antiviral activity and effectively inactivate T4-like phages (7 log reduction) after white light irradiation. One of the aims of this work was to study the possibility of developing the mechanisms of resistance of the viral particles to photoinactivation during APDT. For this purpose, new phage suspensions were obtained after each PDT cycle. After ten cycles of irradiation of phages with white light (28 J/cm^2^), no decrease in the photoinactivation efficiency of the microorganism was observed, hence, it can be concluded that bacteriophages do not possess the ability to acquire resistance to APDT. The same authors studied the mechanism of photoinactivation of phage T4 DNA virus. When singlet oxygen quenchers (sodium azide and L-histidine) were used, their protective effect against APDT was observed, thus confirming the type II photodynamic reaction for the inactivation of phages of this type [45].

Herpes simplex virus (HSV), a member of the Herpesviridae family, affects mammals, birds, reptiles, amphibians, fish, and mollusks [116]. Herpes virus infection has serious complications especially in immunocompromised patients, pregnant women and infants [117]. In the following work the authors studied photodynamic activity of cationic TMPyP and anionic TPPS_4_, as well as their zinc complexes (ZnTMPyP and ZnTPPS_4_) in inactivating bovine herpes virus type 1 (BoHV-1) [118] In dark toxicity studies, none of the samples affected virus viability. However, when BoHV-1 samples were irradiated in the presence of porphyrins, the effectiveness of the studied photosensitizers decreased in the following order: ZnTMPyP > TMPyP > ZnTPPS4 > TPPS_4_. The effect of PDI increased with increasing irradiation dose, and after 120 min, ZnTMPyP, TMPyP, and ZnTPPS_4_ induced complete inactivation of the virus.

Antiviral activity of four asymmetric A3B-type porphyrins **12**–**13a**,**b** (Figure 8) containing combinations of nitro- and pyridyl groups against HIV-1 and SIVmac viruses was studied in the work [119]. The authors investigated the effect of porphyrins on different stages of the virus life cycle under non-photodynamic and photodynamic conditions. No decrease in virus production, release and maturation was observed in the presence of the tested compounds for the already infected cells after 24 h. The studied compounds did not block virus replication once the viral DNA was already transfected. However, at the early stage of the virus life cycle, inhibition of HIV-1 virus penetration was observed upon addition of all the tested compounds, and the maximum effect was achieved with the zinc complex **13b**. Introduction of Zn(II) into the porphyrin macrocycle enhances the antiviral activity of porphyrins.

#### 2.5.2. Cationic Ammonium Derivatives

**Figure 9:**
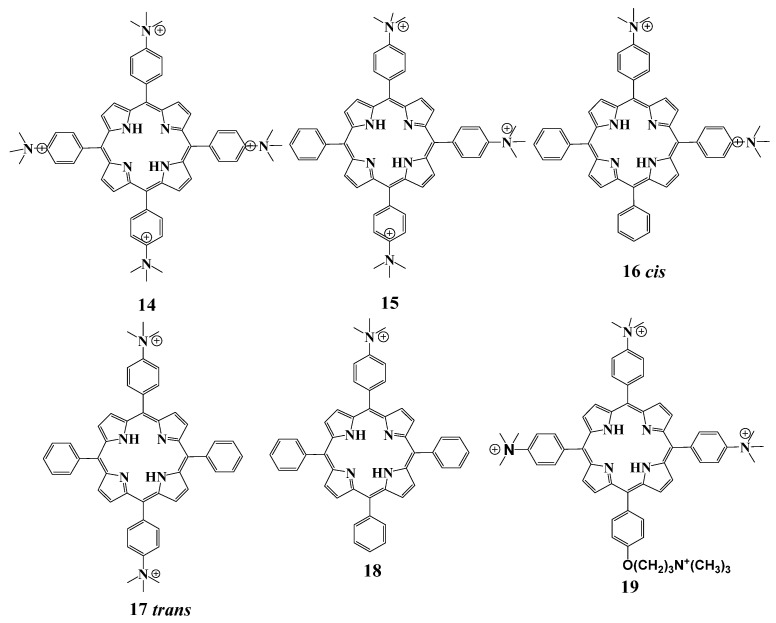
Chemical structure of compounds **14**–**19**.

The next type of cation used in porphyrin chemistry is the substituent based on the ammonium salts. Thus, *meso*-tetrakis(4′-N,N,N-Trimethylammoniumphenyl)porphyrin **14** can be synthesized by the alkylation reaction from the commercially available *meso*-tetrakis(4-aminophenyl)porphyrin (Figure 9). The antimicrobial action of this porphyrin was one of the first examples of photodynamic inactivation observed in *Enterococcus seriolicida*, *Vibrio anguillarum* and *E. coli* [81].

Hurst et al. evaluated the effect of charge in the PS molecule on the PDIs of the Gram-negative bacteria *Escherichia coli* and studied their interaction with the cell membrane [120]. A series of *meso*-substituted porphyrins **14**–**18** containing different number of cationic groups were obtained (Figure 9). It was found that monocationic porphyrin **18** did not inactivate *E. coli* cells under irradiation below the concentration of 100 μM. The individual effect of dicationic porphyrins **16**–**17** represented as a mixture of cis and trans isomers was not possible to assess. Tetra- and tricationic porphyrins **14**–**15** were the most effective PSs providing complete inactivation of *E. coli* cells in the concentration range of 1–10 μM. Investigation of the PS localization in prokaryotic cells was carried out using fluorescence microscopy. These studies showed that compounds **15**–**18** localize outside the *E. coli* membrane, while porphyrin **14** penetrates the cell membrane. The nature of interaction of **14** with *E. coli* cells depends on the incubation time, since at incubation time less than 15 min porphyrin **14** localizes also on the membrane surface. Authors suggested that compound **14** may interact with lipopolysaccharides of the outer membrane of bacteria, which leads to the formation of pores and subsequent internalization of the porphyrin molecules by the cells.

Tetracationic porphyrin **14** and its tricationic analogue **19** were tested for inactivation of *E. coli* immobilized on an agar surface. This system can serve as a prototype for the local areas of infection on the skin. Compound **19** demonstrates higher photostability compared to **14** and higher photoinactivating efficiency towards microbes immobilized on agar [121].

**Figure 10:**
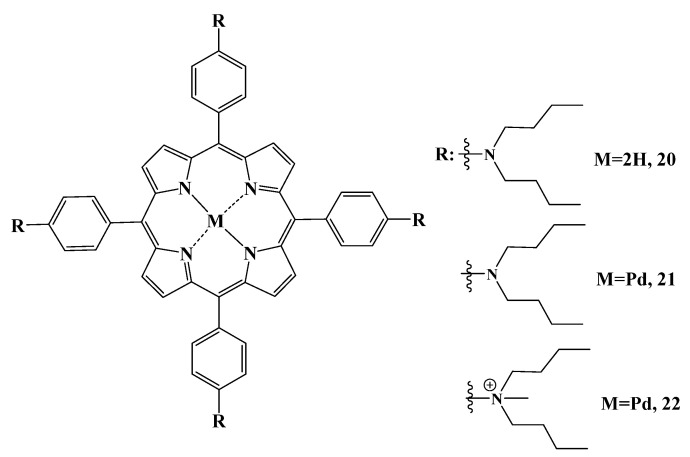
Chemical structure of compounds **20**–**22**.

Tetra-*meso*-dibutylaminophenylporphyrin **20**, and its complex with palladium **21** and palladium complex of tetra-cationic porphyrin **22** (Figure 10), were prepared in the work [122]. The obtained PSs were immobilized with chitosan. Chitosan is a biocompatible and biodegradable biomaterial with wound healing properties [123]. It was also used as a drug delivery vehicle [124]. Chitosan immobilized with porphyrin **22** was shown to provide 100% bacterial cell death under light activation against *S. aureus* after 15 min of irradiation. Free porphyrin **20** achieved this effect only after 30 min of irradiation. Apparently, this phenomenon is associated with the additional antibacterial effect of chitosan. Neutral photosensitizers **20**–**21** and their complexes with chitosan had high singlet oxygen quantum yields in DMSO, but showed low photodynamic activity associated with their aggregation in aqueous biological media.

#### 2.5.3. Cationic Imidazolium Derivatives

Imidazole derivatives are known to possess antimicrobial, fungicidal and cytotoxic activities [125]. In the Neves group, approaches to the preparation of new imidazole-containing porphyrins were developed, and their antimicrobial photodynamic activity as well as the influence of KI addition on the APDT procedure, were investigated [126,127]. Potassium iodide upon interaction with ^1^O_2_ forms free iodine (I_2_/I_3_^−^), hydrogen peroxide (H_2_O_2_), and iodine radicals (I_2_^−^), which are highly bactericidal [127]. To evaluate the photoinactivation activity of each PS against *E. coli*, the compounds were tested at different concentrations (5.0 and 20.0 μM) in the absence and in the presence of 100 mM KI. As a result of photochemical studies, compounds **23c** and **23d** showed less ability to generate ^1^O_2_ compared with **23a** and **23b**, which accounted for the slow photoinactivation of bioluminescent *E. coli* both in the presence and in the absence of KI. The authors also mention that when derivative **23b** was used with KI, the bacterial inactivation curve underwent a sharp decrease in the first 15 min of irradiation, indicating a significant enhancement of its photodynamic effect in the presence of potassium iodide. Because of the sharp decrease in the survival profile of *E. coli*, it was concluded that the mechanism of action of the porphyrin-imidazole derivative **23b** + KI combination is probably related to the predominant decomposition of peroxyiodide into free iodine (I_2_/I_3_^−^).

**Figure 11:**
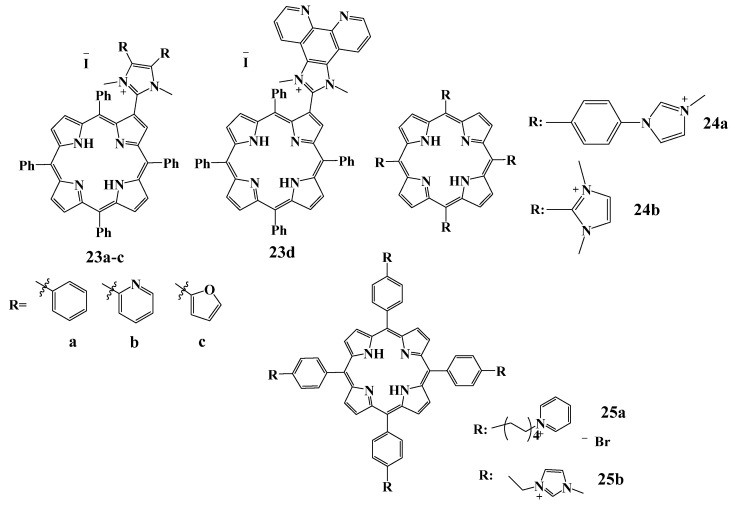
Chemical structure of the cationic imidazole porphyrins.

The authors of [128] conducted studies on the porphyrins with phenylimidazole and methylimidazole groups **24a**,**b** and TMPyP for photoinactivation of three types of bacteria: *S. aureus*, *E. coli* and *Pseudomonas aeruginosa* (Figure 11). All the porphyrins tested were effective against these strains upon irradiation, but there were differences in the minimum bactericidal concentrations (MBC). Thus, compound **24b** (25 and 40 µM against *E. coli* and *Pseudomonas aeruginosa*, respectively) was found to have the lowest efficacy and the highest MBC. This effect seems to be associated with the small size of the imidazole *meso*-substituent and the higher positive charge density. Porphyrin **24a** was the most effective against *E. coli*, thus, its MBC was seven times less than that of the known TMPyP.

**Figure 12:**
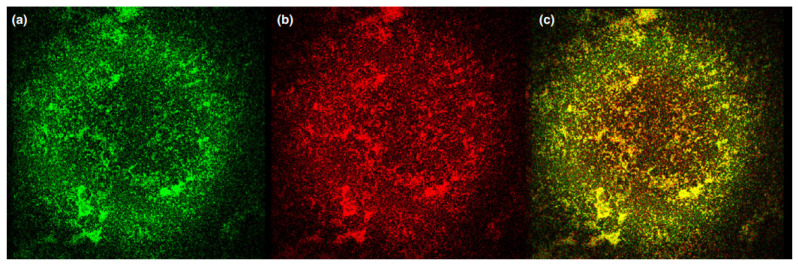
*Aggregatibacter actinomycetemcomitans* bacterial biofilm with **25b** porphyrin. (**a**) Green autofluorescence, λex: 488 nm; λem: 500–600 nm, (**b**) red porphyrin fluorescence, λex: 635 nm; λem: 650–720 nm and (**c**) the overlap of both (**a**,**b**). Objective: ×63. Copyright 2014 WILEY [129].

**Figure 13:**
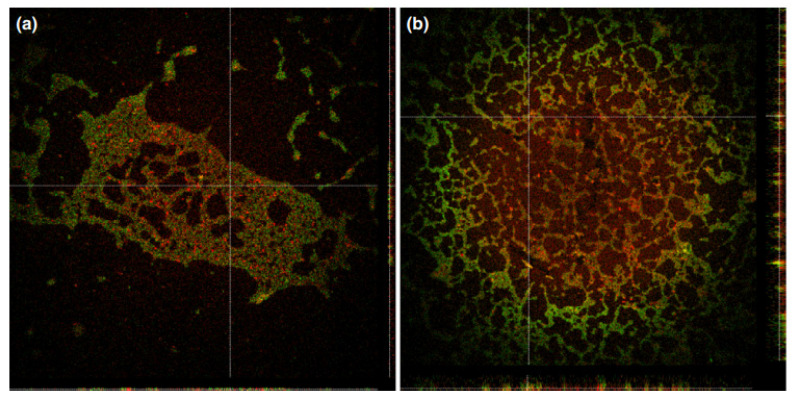
Confocal laser scanning microscope images of *E. faecalis* biofilms incubated with **25b** (**a**) and **25a** (**b**). The z-profile of the biofilms shows the thicknesses between 4 and 8 lm as seen on the right and bottom position inside the images. Copyright 2014 WILEY [129].

Cationic porphyrins **25a**,**b** [129] showed efficacy in killing bacteria causing dental pathologies, reducing the cell survival by more than 99.9% after light. Porphyrin **25a** with pyridinium residues at the concentration of 22 µM showed no dark toxicity against Gram-negative *A.actinomycetemcomitans*, *F. nucleatum* and Gram-positive *E. faecalis*, whereas the meta-imidazole derivative **25b** exhibited dark toxicity for the whole range of concentrations tested (2–22 µM). Optimal photoinactivation of *E. faecalis* cells by imidazole-substituted porphyrin **25b** was observed at very low (2 µM) concentration of the photosensitizer. In the same work, the uptake and localization of porphyrins in the planktonic bacteria and artificially grown biofilms were evaluated using fluorescence measurements of the cell extracts and direct imaging by confocal microscopy. The results suggested that both cationic porphyrins, due to their positive charges, readily attach to the cell wall through noncovalent electrostatic interactions [130] and the target for the generated singlet oxygen is the membrane of pathogens. In the biofilm slice image, where the green color represents the autofluorescence of the bacterial biofilm of *A. Actinomycetemcomitans* and red represents the fluorescence of porphyrin in the range of 650–720 nm, under laser excitation at 635 nm, the heterogeneous structure of the biofilm studied is clearly observed, as well as the fluid-filled channels and voids. The vertical slice image of *A. actinomycetemcomitans* biofilm shows that the cationic porphyrins were unable to penetrate the high-cell-density regions, which can be explained by the intact anaerobic internal clusters formed within the biofilm. However, in the case of *E. faecalis* biofilms, both porphyrins were more evenly distributed due to their loose structure (Figure 12 and Figure 13).

#### 2.5.4. Other Cationic Derivatives

The last group of compounds has a different structure of cationic substituents; therefore, we have referred all these compounds to a separate group. In [131] the authors report the photodynamic activity of cationic β-vinyl substituted *meso*-tetraphenylporphyrins **26a**,**b** against the herpes simplex virus (HSV-1). Virucidal activity tests were performed by exposing HSV-1 suspensions to the highest non-cytotoxic concentration of PS obtained in photocytotoxicity assays (0.5 µM). The degree of virus inactivation upon the exposure time was also evaluated. The results showed that both compounds had an inhibitory effect on the infectivity of the virus. However, only compound **26b** exhibited virucidal activity under dark conditions (28% viral inactivation). Nevertheless, this activity was strongly enhanced upon PS activation with light: virus inactivation reached 97% after 5 min of irradiation and 99% when the photoactivation time was increased to 15 min. Compound **26a** has also exhibited an antiviral effect, but only when exposed to light, and apparently had a delayed onset of action, since virus inactivation occurred only after 15 min and only in half of the pathogen population.

**Figure 14:**
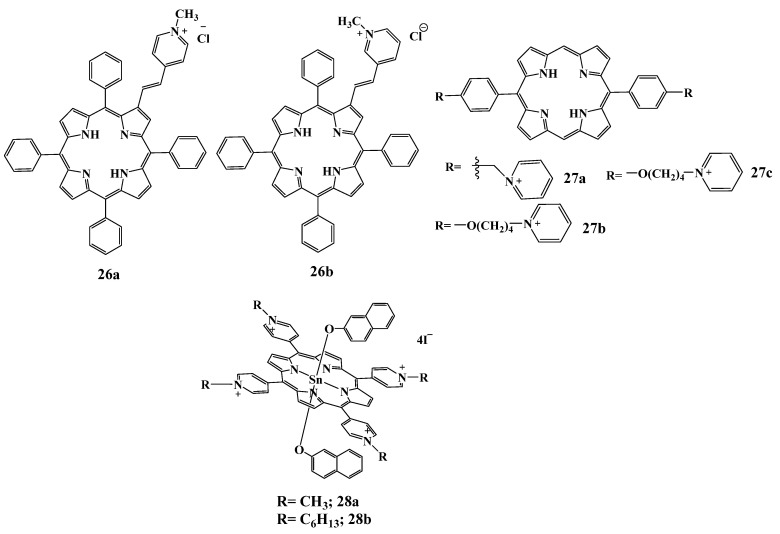
Chemical structure of the compounds **26**–**28**.

The influence of the charge arrangement in di-cationic porphyrins of ABAB-type **27a**–**c** was demonstrated in [132]. Porphyrins **27b**,**c** (Figure 14), containing long-chain pyridinium substituents (four and eight methylene links, respectively) at 5,15-positions of the tetrapyrrole macrocycle, were toxic to *P. aeruginosa*, while **27a**, bearing a benzyl chain at the same positions of the pyridinium group, had no toxic effect. It was suggested that the positive charge of compound **27a** is highly delocalized since the pyridinium nucleus is directly conjugated to the tetrapyrrole system of the porphyrin. The uniform charge distribution should prevent the interaction of diarylporphyrins with anionic components of the bacterial cell wall. Conversely, the positive charge located at the periphery of the macrocycle at **27b**,**c** can favor a strong electrostatic interaction with the bacterial cell wall. This interaction can be sufficient to destroy the integrity of the cell wall, decreasing bacterial viability irrespective of irradiation.

Balaji Babu et al. [133] obtained two cationic porphyrins **28a**,**b** with 2-naphthalate transoxy ligands (Figure 14). Moreover, the compounds differed in lipophilicity; hexyl groups were attached to quaternized nitrogen atoms in the structure of **28b**. The obtained compounds had high values of singlet oxygen quantum yield (0.90) and fluorescence lifetime. Both compounds exhibited dark toxicity against *S. aureus*. After 15 min of irradiation (250 mW/cm^2^), **28a** reduced cell viability to <1%, whereas **28b** resulted in the destruction of 91% of viable cells. After 90 min of irradiation, cell viability decreased to 16.3% and 0% for **28b** and **28a**, respectively. Thus, the introduction of four hexyl substituents significantly increased the hydrophobicity of the photosensitizer and decreased its efficiency as a PS due to the increased tendency to aggregation in polar medium.

**Figure 15:**
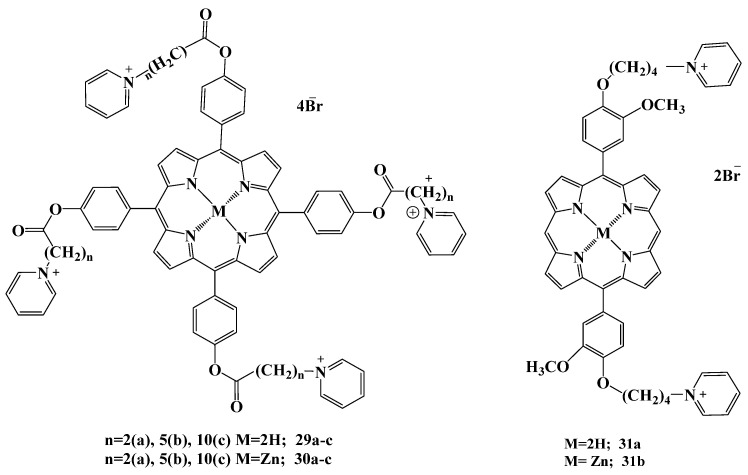
Chemical structure of compounds **29**–**31**.

In our studies [134,135,136,137], we investigated the antimicrobial activity against *S. aureus* and *E. coli* bacteria and their biofilms for cationic *meso*-arylporphyrins with A4 and ABAB structure with terminal pyridinium groups (Figure 15). In these works, we modified the following parameters in the structure of the compounds: (1) the hydrophilic–hydrophobic balance varied depending on the number of methylene spacers (2, 4, 5, 10); (2) the presence of the central Zn (II) atom; (3) the use of micellar delivery vehicles based on Pluronic F127.

**Figure 16:**
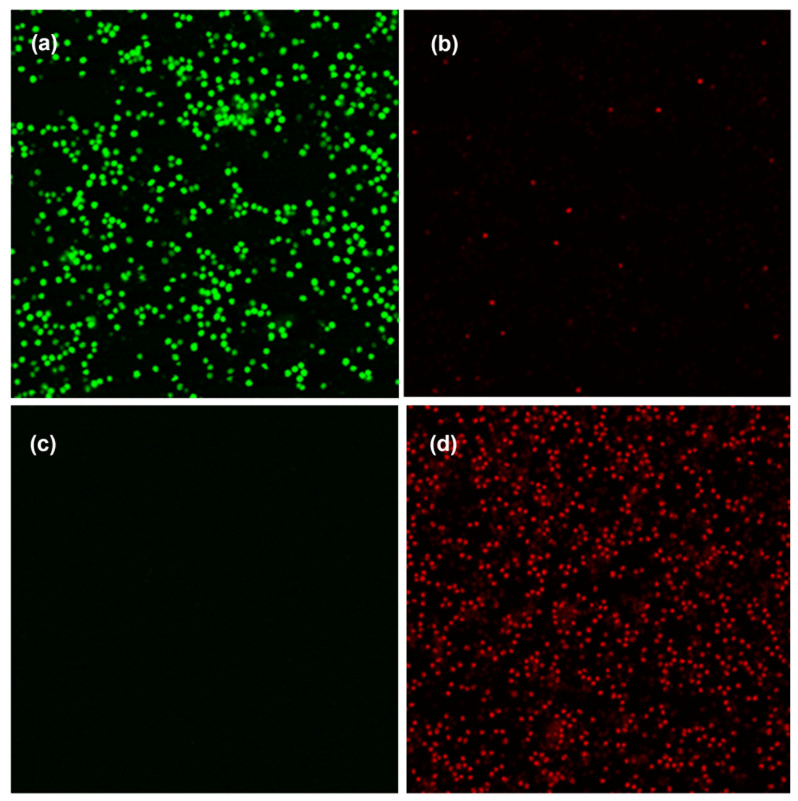
Bactericidal effect of **29b** is related to permeabilization of the bacterial membrane. Confocal images of *Staphylococcus aureus* bacterial biofilms, stained by BCECF and PI: intact cells (**a**,**c**) and cells incubated with 10 μM of **29b** and irradiated (**b**,**d**). (**a**,**c**) BCECF fluorescence in the 500–550 nm spectral range. (**c**,**d**) Fluorescence of PI in the 650–700 nm range. The bar size is 6 μm. Images were recorded in similar conditions and can be compared by signal intensity. BCECF: 20, 70-Bis-(2-carboxyethyl)-5-(6)-carboxyfluorescein; PI: Propidium iodide. Copyright 2020 Elsevier [134].

We have shown that the efficiency of the used PS decreases with the increasing length of the methylene spacers, which is associated with an increase in their hydrophobicity, and hence with the aggregation processes in aqueous media. Therefore, we decided to use a micellar drug delivery system based on Pluronic F127. Previously, this biodegradable polymer was used for delivery of PS into cells. The use of Pluronic reduces the recognition by reticuloendothelial system and increases the time of PS circulation in the blood [138,139]. The application of Pluronic as a delivery vehicle for PS had a positive effect on antibacterial activity against *S. aureus* for compounds **29a**–**c** and **30a**,**b**, with the dose of PS reduced by four to eight times. Thus, the MIC after irradiation for compound **29a** in PBS/DMSO solution (*v*/*v* 10:1) decreased from 20 to 0.6μM, from 10 to 2.5 μM for **29b** and from 5 to 0.6 μM for **29c**. The MIC was not reached for compounds **30a**,**b** in DMSO in the studied concentration range (0.6–40 μM); however, MIC amounted to 10 μM in presence of Pluronic. In the study of photoinduced effect of PS on *S. aureus* bacterial biofilms, 100% inhibition of viable bacteria was recorded for compounds **29**–**30a**,**b** at concentrations of 10 μM. The maximal degree of inhibition (82%) was achieved for zinc complex **30b** against *E. coli* biofilms [134,135]. The mechanism of photodynamic action against bacterial biofilms of Gram-positive *S. aureus* strain was investigated by confocal laser microscopy [134]. Incubation of bacteria with hydrophobic acetoxymethyl ester of BCECF leads to intracellular accumulation of this dye followed by its cleavage by cellular esterases to form highly charged fluorescent carboxyfluorescein dye (BCECF) (Figure 16). The intact cell membrane is impermeable for propidium iodide (PI) added to the cells as well as to the intracellular charged BCECF. Treatment of bacteria with the solution of cationic compound **29b** (10 μM for 1 h) followed by irradiation resulted in leakage of BCECF from the cells and accumulation of intracellular PI, indicating bacterial cytoplasmic membrane damage caused by PS. Thus, the CLSM data showed that **29b** acts on Gram-positive bacteria through their plasma membrane components damage during irradiation.

The antiviral activity of compounds **29a**,**b** against the herpes simplex virus 1 (HSV-1) was also evaluated in our work [137]. Compounds **29a**,**b** and their Pluronic F-127 micellar forms showed virucidal efficiency and affected the different replication stages of HSV-1. TMPyP was also used as a reference compound. Cationic porphyrin **29b** showed the highest antiviral activity, close to 100% at the lowest concentration of 3.6 µM, while the maximum of TMPyP activity was observed with a concentration of 13.3 µM; porphyrin **29a** as the most hydrophilic compound was the least active. Solubilization of the synthesized compounds in Pluronic F-127 polymeric micelles had a noticeable effect on the antiviral activity only at higher porphyrin concentrations. The action of the obtained compounds differs by the influence on the early or later reproduction stages. While **29a** and TMPyP acted on all stages of the viral replication cycle, porphyrin **29b** inhibited virus replication during the early stages of infection.

*Trans*-A2B2 type dicationic porphyrins **31a**,**b** in the form of free base and zinc complex [136] were used to study the effect of the PS structure on photodynamic antibacterial activity. In the study of the photoinduced antibacterial activity of these porphyrins against *Staphylococcus aureus* strain 78 zinc complex **31b** was found to exhibit photodynamic action within the whole concentration range studied, even at a minimum irradiation dose of 2.3 J/cm^2^ compared to the free-base porphyrin, which showed lower efficiency even at the maximum irradiation dose. The obtained results correlate with the photochemical activity data of the target compounds.

### 2.6. Approaches to Improve Antibacterial Effect of PS

#### 2.6.1. Development of Antimicrobial Surfaces

**Figure 17:**
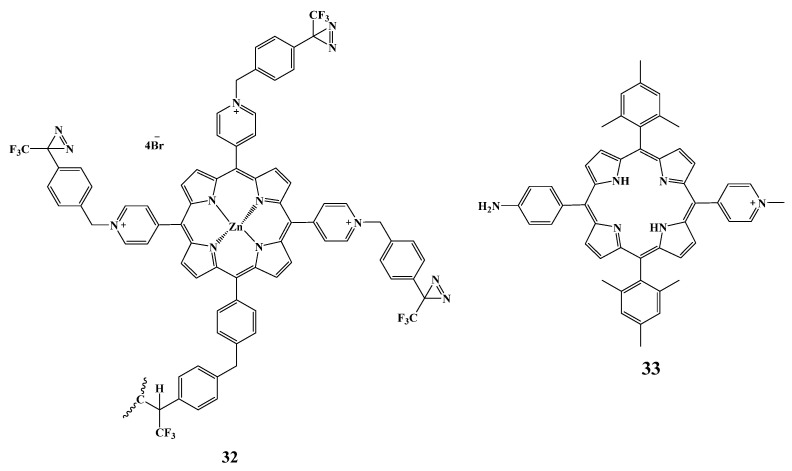
Chemical structure of the compounds under investigation.

One of the approaches to controlling resistant strains of bacteria and infection using biofilms formed by pathogens is the development of photoactive self-sterilizing surfaces. The research [140] evaluated the antimicrobial effect of photoactive polyethylene terephthalate (PET) surfaces covalently functionalized with cationic diazirine-containing (diazirine) porphyrin **32** (Figure 17) against the growth of *E. coli* ATCC 25922 and *Pseudomonas aeruginosa* (ATCC^®^ 10145™). Irradiation with white LED light for 6 h resulted in 97 ± 0.3 and 99.95 ± 0.02% reduction of planktonic *P. aeruginosa* and *E. coli*, respectively. The study also evaluated the effect of functionalized PET discs on biofilm formation by *E. coli*, *P. aeruginosa* and *S. aureus* (ATCC^®^ 6538P™). Scanning electron micrographs demonstrated morphological changes in *S. aureus* cells grown on porphyrin-functionalized surfaces after light exposure, while morphological changes in *P. aeruginosa* and *E. coli* were less obvious. Electron micrographs also showed that exposed-to-light biofilms formed on PET discs were reduced to cell clusters characterized by a less complex three-dimensional structure and lower surface coverage compared to the bacterial strains grown outside the photoactive surface. Confocal images as well as biovolume measurement data showed that the number of biofilms, formed on irradiated functionalized PET surfaces, was reduced by 68 ± 5%, 73 ± 8% and 78% ± 3% for *S. aureus*, *P. aeruginosa* and *E. coli*, respectively.

The work [141] focuses on the development of cellulosic cotton fibers containing three types of PSs—cationic **33** (Figure 17), anionic TPPS4 and neutral TPP-NH2 for antimicrobial applications. The PSs were covalently attached to the cotton fabric using a triazine linker. The antimicrobial activity of porphyrin-cellulose materials was tested under visible light irradiation against *S. aureus* and *E. coli*. For *S. aureus*, the percentage of bacterial growth inhibition is 37% for the anionic cotton material, 93.7% for neutral cotton and 100% for cationic cotton. No photodamage was observed for Gram-negative *E. coli*.

#### 2.6.2. Preparation of Conjugates with Antibiotics

**Figure 18:**
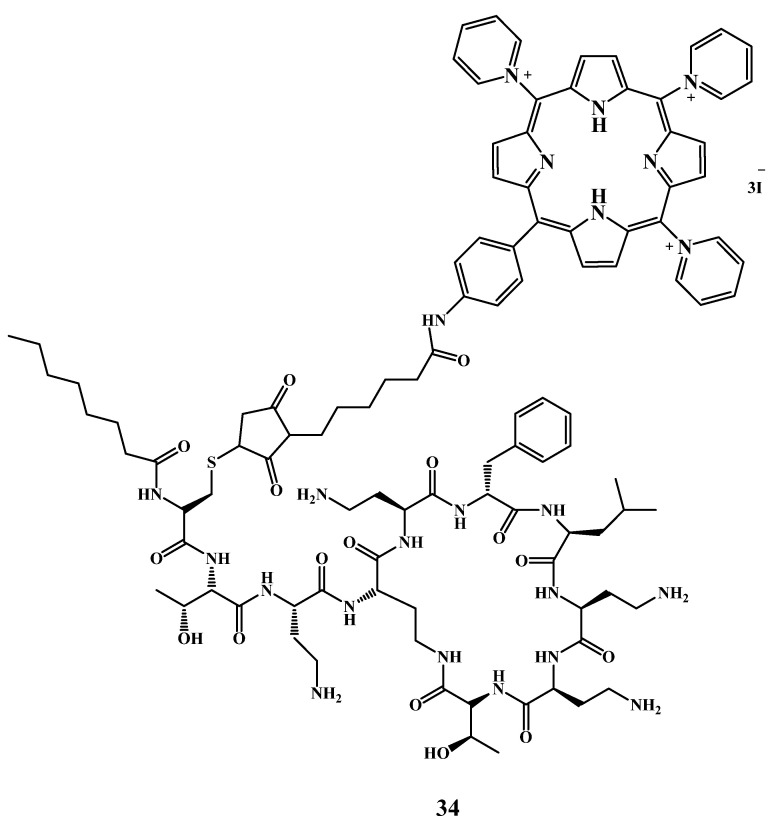
Structure of the cationic porphyrin−polymyxin B conjugate.

Conjugation of the PS with antibiotics is a new synergistic approach to inactivate bacteria. Such an approach can significantly improve the photodynamic antibacterial activity since the antibiotics provide targeting of the outer membrane of Gram-negative bacteria. A cationic porphyrin covalently attached to polymyxin B derivative (Figure 18) was synthesized by Le Guern et al. [141]. Conjugate **34** exhibited enhanced activity and targeting compared to the unsubstituted porphyrin analog and TMPyP used as a reference compound. PS **34** was active upon irradiation against *S. aureus*, *P. aeruginosa* and *E. coli*. Its MIC was 0.5 µM, whereas the MIC of TMPyP was 18 µM.

#### 2.6.3. Preparation of Conjugates with Nanoparticles

Currently there is a demand to develop new low-cost materials for in situ controlled and safe delivery of PSs to the site of bacterial infection. Nanoparticles are widely used to improve PS performance in photomedicine [142,143]. The study [144] is devoted to encapsulating TMPyP into PLGA nanoparticles to create topical hydrogels. Penetration of the cationic PS was not observed through the skin of the pig ear and histologic examination did not detect any damage in the surrounding tissues after APDT.

The design and characterization of a nanophototherapeutic agent based on commercially available cyclodextrin Captisol (sulfobutyl ether-β-cyclodextrin) and tetra(p-toluene sulfonate) TMPyP to create effective biocompatible systems for application in APDT is proposed in the work of Zagami et al. [145]. Spherical nanoparticles of about 360 nm in size based on Captisol/TMPyP supramolecular complexes with 1:1 stoichiometry were prepared by stirring in aqueous medium and freeze drying. Release and photostability studies were performed under physiological conditions. Captisol was found to contribute to the maintenance of porphyrin release for more than 2 weeks and to protect the PS from photodegradation. The antimicrobial activity of the nanoassemblies was investigated against Gram-negative *P. aeruginosa*, *E. coli* and Gram-positive *S. aureus*. The proposed nanosystems were able to photoinhibit both Gram-positive and Gram-negative bacterial cells similarly to free TMPyP. The Captisol/TMPyP system demonstrated delayed release properties and enhanced photostability, thus optimizing the effect of PDT at the site of action.

#### 2.6.4. Preparation of Conjugates with Antimicrobial Peptides

Antimicrobial peptides (AMPs) are considered one of the most promising alternatives to antibiotics. AMPs, also called host-defense peptides, are part of the immune system in bacteria, plants, and animals [146,147,148]. AMPs were studied against Gram-positive and Gram-negative bacteria, as well as fungi, parasites, and viruses [146]. Conjugation of AMPs that specifically target bacterial cells with PS is a promising synergetic strategy to enhance antimicrobial activity. Recently several studies have demonstrated the perspective of AMPs-PS conjugates. Thus, conjugates of different AMPs with purpurin 18 [149], chlorin e6 [150], 5-(4-carboxyphenyl)-10,15,20-triphenylporphyrin [151] were studied.

**Figure 19:**
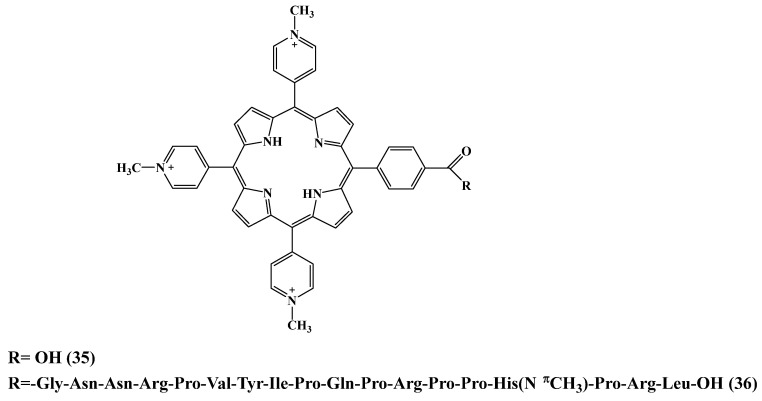
Structure of the cationic porphyrin−AMP conjugate [152].

Dosselli et al. [152] reported a series of novel antimicrobial peptide apidaecin-porphyrin conjugates for *E. coli* and methicillin-resistant *S. aureus* (MRSA) inactivation (Figure 19). The structure of the PS consisted of neutral or cationic charged porphyrin **35**–**36** or porphycene. In this study, unfortunately, apidaecin’s targeting properties were lost after conjugation to a bulky PS. The use of another treatment protocol including repetitive washing after sensitizer delivery improved the activity of conjugate **36** compared to the apidaecin-free porphyrin **35**. Conjugate **36** caused total photokilling of *E. coli* cells at a concentration (10 μM). Despite the controversial result of this work, the creation of AMPs-PS conjugates requires further investigation.

## 3. Conclusions

The resistance of microorganisms to known antibiotics, the growth of new bacterial and viral infections and public biosafety issues, including food and water safety, require new strategies to suppress bacterial and viral infections. Numerous works published in the last decade show an increasing interest towards APDT as an alternative tool to the traditional therapy, as the generated ROS do not induce resistance. Multitarget action of ROS including membrane lipids, proteins and nucleic acids is the main advantage compared to antibiotic or antiviral drugs, which affect a single target. Most of the studies are focused on the synthesis of effective PSs and examination of their biological activity. New approaches to improving the efficiency of APDT and the creation of new antibacterial materials are being proposed.

Tetrapyrrole compounds exhibit a broad spectrum of antiviral and antibacterial activities, which makes them promising compounds for the development of new PSs for APDT. This review summarizes the available results of the chemical structure–antimicrobial activity relationship for cationic porphyrin derivatives. Positively charged porphyrin derivatives have several advantages and are considered the most suitable PSs for APDT. They possess a high affinity towards bacterial membranes, chemical stability, have a low cost and a well-developed chemistry. This allows for the directed preparation of porphyrin moieties with the required set of chemical substituents in the macrocycle. Tetrapyrroles with cationic groups are good candidates for broad-spectrum antimicrobial agents.

Biological activity of cationic porphyrins against microorganisms can be determined by a number of factors: asymmetric substitution system in the macrocycle (porphyrins of A3B type show increased antimicrobial activity), amphiphilicity of the molecule (presence of hydrophobic substituents along with cationic groups in one molecule), presence of central metal atom in the macrocycle or external chelation at the periphery of the macrocycle (varying the metal atom makes it possible to increase the antimicrobial effect), conjugation with biologically active molecules (antibiotics, peptides, antiviral drugs, etc.) and conjugation with various nanoparticles. The establishment of antimicrobial activity mechanisms is of great practical interest for the development of new PSs, but this area of research to date is mostly at the stage of accumulation and generalization of the experimental data. These studies should include the peculiarities of the structure and development of the individual microorganisms, especially in the case of viruses. Such new aspects undoubtedly include the light-independent antimicrobial activity of tetrapyrroles, which in the future can be considered a reliable option for systemic application of APDT. The above data show the demand for a continued search for new classes of PSs for APDT.

## Data Availability

Data are contained within the article.
